# Recognition of Human Erythrocyte Receptors by the Tryptophan-Rich Antigens of Monkey Malaria Parasite *Plasmodium knowlesi*

**DOI:** 10.1371/journal.pone.0138691

**Published:** 2015-09-22

**Authors:** Kriti Tyagi, Deepali Gupta, Ekta Saini, Shilpa Choudhary, Abhishek Jamwal, Mohd. Shoeb Alam, Mohammad Zeeshan, Rupesh K. Tyagi, Yagya D. Sharma

**Affiliations:** Department of Biotechnology, All India Institute of Medical Sciences, Ansari Nagar, New Delhi 110029, India; Université Pierre et Marie Curie, FRANCE

## Abstract

**Background:**

The monkey malaria parasite *Plasmodium knowlesi* also infect humans. There is a lack of information on the molecular mechanisms that take place between this simian parasite and its heterologous human host erythrocytes leading to this zoonotic disease. Therefore, we investigated here the binding ability of *P*. *knowlesi* tryptophan-rich antigens (PkTRAgs) to the human erythrocytes and sharing of the erythrocyte receptors between them as well as with other commonly occurring human malaria parasites.

**Methods:**

Six PkTRAgs were cloned and expressed in *E*.*coli* as well as in mammalian CHO-K1 cell to determine their human erythrocyte binding activity by cell-ELISA, and in-vitro rosetting assay, respectively.

**Results:**

Three of six PkTRAgs (PkTRAg38.3, PkTRAg40.1, and PkTRAg67.1) showed binding to human erythrocytes. Two of them (PkTRAg40.1 and PkTRAg38.3) showed cross-competition with each other as well as with the previously described *P*.*vivax* tryptophan-rich antigens (PvTRAgs) for human erythrocyte receptors. However, the third protein (PkTRAg67.1) utilized the additional but different human erythrocyte receptor(s) as it did not cross-compete for erythrocyte binding with either of these two PkTRAgs as well as with any of the PvTRAgs. These three PkTRAgs also inhibited the *P*.*falciparum* parasite growth in in-vitro culture, further indicating the sharing of human erythrocyte receptors by these parasite species and the biological significance of this receptor-ligand interaction between heterologous host and simian parasite.

**Conclusions:**

Recognition and sharing of human erythrocyte receptor(s) by PkTRAgs with human parasite ligands could be part of the strategy adopted by the monkey malaria parasite to establish inside the heterologous human host.

## Introduction

The monkey malaria parasite *Plasmodium knowlesi* has emerged as a potential threat to humans [1, 2]. To infect and grow inside the heterologous host, the *P*.*knowlesi* molecules should be able to recognize the receptors on the human erythrocytes. One such common molecule present on monkey and human erythrocytes involved in invasion process by *P*.*knowlesi* has been identified as Duffy Antigen [3, 4]. Duffy antigen independent binding of *P*.*knowlesi* ligand called PkNBPXa to human erythrocytes has also been described in the literature [5]. Nevertheless, the red cell invasion by the parasite requires larger repertoire of host and parasite molecules. Therefore, it is important to identify such key proteins for the effective development of therapeutics.

Tryptophan-rich proteins were first described from murine malaria parasite *P*.*yoelii* where they showed erythrocyte binding activity as well as partial protection in mice against this parasite [6]. Later on, these proteins were described from human and simian malaria parasites [7–12]. Peptides derived from *P*.*falciparum* tryptophan—threonine rich antigen (PfTryThrA) have been shown to block invasion of human erythrocytes by this parasite [13] while over expression of another tryptophan-rich protein called PArt has been implicated in artesunate tolerance [7]. As compared to *P*.*yoelii* and *P*.*falciparum*, the *P*.*vivax* parasite contains larger number of tryptophan-rich antigens belonging to ‘Pv-fam-a’ family [10]. Earlier, we have reported that ten out of 36 *P*.*vivax* tryptophan-rich antigens (PvTRAgs) show erythrocyte binding capability [14, 15]. It was hypothesized that the erythrocyte binding PvTRAgs which are expressed at the late stage of the parasite are probably associated with red cell invasion while those expressed at early stage could be involved in rosetting phenomenon [15–18]. Rosetting is observed in malarial patients where several of the uninfected erythrocytes bind to a single parasitized RBC. These rosettes block the normal blood flow in capillaries leading to disease severity [19]. These reports suggest the biological significance of *Plasmodium* tryptophan-rich proteins and their potential as drug/vaccine targets.

Simian malaria parasite *P*. *knowlesi* also contains a large number of tryptophan-rich antigens which are mostly expressed during the blood stages of the parasite [11, 20]. It would be interesting to know if *P*.*knowlesi* tryptophan-rich antigens (PkTRAgs) are also capable of interacting with human RBCs, using same or different erythrocyte receptors utilized by *P*.*vivax* and *P*.*falciparum* ligands. This would enable us to understand the hitherto unknown molecular mechanisms that are taking place during host-parasite interaction in a heterologous system of this zoonotic disease [5, 14, 15]. Here, we show that PkTRAgs bind to human erythrocytes, utilizing same as well as different erythrocyte receptors as that of the PvTRAgs of *P*.*vivax*, and were also able to inhibit the *P*.*falciparum* parasite growth in in-vitro culture.

## Methods

### Ethics statement

Heparinized blood was collected from healthy individuals following the Institutional ethical guidelines. The written consent was obtained from the individuals prior to blood collection. Ethics committee of All India Institute of Medical Sciences, New Delhi had approved the study via approval number IEC/NP-342/2012 & RP-11/2012. All animal experiment protocols were approved by the Institutional Animal Ethics Committee (Approval number 494/IAEC/09). The ICMR and GCP guidelines were followed to perform human studies and CPCSEA guidelines were followed to perform experiments on animals.

### Cloning and expression of recombinant PkTRAgs in *E*.*coli*

The exon 2 of PkTRAg38.3, PkTRAg44.7, PkTRAg40.1, PkTRAg67.8, and only tryptophan-rich domain (TRD) of PkTRAg67.1 and PkTRAg88.2 were PCR amplified from genomic DNA of *P*.*knowlesi* (a kind gift from Dr. Chetan Chitnis) using primers and PCR conditions given in S1 Table. PCR products were cloned into pGEM^®^-T easy (Promega corporation, Madison, WI, USA) and then sub cloned into the PproEx HTa/b/c vectors (Invitrogen Life Technologies, Carlsbad, CA, USA). Expression and purification of the recombinant histidine-tagged PkTRAgs using Ni^2+^ NTA resin was carried out as described elsewhere [21]. Proteins were checked on 12% SDS-PAGE and confirmed by western blot analysis using monoclonal anti-His_6_ antibody (Sigma-Aldrich, St. Louis, MO, USA).

### Expression of PkTRAgs on the surface of CHO-K1 cells

Exon 2 or TRD of PkTRAgs from the above recombinant plasmids were cloned into *PvuII* and *ApaI* sites of pRE4 vector (kind gift from Dr Gary Cohn and Dr Roselyn Eisenberg) in frame with the signal sequence and transmembrane segment of the *Herpes simplex* glycoprotein D (HSVgD) [22]. CHO-K1 cells were transfected with recombinant plasmid by following the same method as described earlier [15]. Expression of PkTRAgs on the surface of transfected CHO-K1 cells was determined by immunofluorescence assay using mouse monoclonal antibodies DL6 (Santa Cruz Biotechnology, Texas, USA) directed against amino acids 272–279 of HSVgD [22, 23]. For secondary antibody, Alexa flour 488 conjugated goats’ anti-mouse antibodies were used. Cells were mounted in mounting solution containing Fluoroshield^TM^ with DAPI (4', 6- diamidino-2-phenylindole a nuclei stain) (Sigma-Aldrich, St. Louis, MO, USA). Images were obtained under Nikon eclipse 80i fluorescence microscope (Nikon Corporation, Tokyo, Japan) at 400x magnification.

### Raising polyclonal antibodies

Purified recombinant PkTRAg38.3, PkTRAg40.1 and PkTRAg67.1 proteins were used for raising antibodies in rabbits. Three hundred micrograms of recombinant fusion protein was emulsified with Freund’s complete adjuvant and injected subcutaneously. After three weeks, three consecutive boosters were given at 2 week intervals each, with 110 μg of recombinant protein emulsified with Freund’s incomplete adjuvant. Ten days after the last immunization, sera were collected from clotted blood, and stored at -20°C.

### Erythrocyte binding assay by Cell-ELISA

ELISA based erythrocyte binding assay was performed as described earlier [15, 24]. Briefly, each well of a 96-well ELISA plate received ~1 million erythrocytes and plate was incubated overnight at 4°C. Next day, the erythrocytes were fixed with 0.3% glutaraldehyde at 25°C for 30 min. The plates were blocked with 5% BSA for 2 h at 37°C. After washing, the plates were incubated with different concentrations (0–2 μM) of recombinant proteins for 4 h at room temperature. Plates were washed with PBST (Phosphate Buffer Saline with 0.05% Tween 20) and incubated with 1: 2000 dilution of the primary mouse monoclonal anti-His_6_ antibody (Serotech, Raleigh, NC, USA), followed by horseradish peroxidase conjugated anti-mouse IgG secondary antibody (Pierce Biotechnology Inc., Rockford, IL, USA). Finally, plates were developed with o-phenyldiamine dihydrochloride substrate (Sigma-Aldrich, St. Louis, MO, USA) and OD was measured at 490 nm. Histidine-tagged PvTRAg35.2 of *P*.*vivax* and thioredoxin from *Desulfovibrio desulfuricans* were used as positive and negative controls, respectively [25].

For competitive inhibition studies, the histidine tag of the recombinant PkTRAgs was first removed by the treatment of AcTEV protease using the manufacturer’s protocol (Invitrogen Life Sciences, Carlsbad, CA, USA). A fixed amount of histidine-tagged PkTRAgs (200 nM) were mixed with increasing concentration (0–2 μM) of respective untagged PkTRAg. This mixture was then added to a 96 well ELISA plate already containing ~1 million erythrocytes and incubated for 4 h at room temperature. After washing with PBST, plate was processed as above. For positive control, no untagged PkTRAg (only PBS) was pre-incubated with respective histidine-tagged PkTRAg.

For cross-competition assay, two μM of untagged protein was added to the ~ 1 million human erythrocytes in a 96 well ELISA plate. After incubating for 4 h at room temperature, plate was washed with PBS and incubated with 1μM of histidine-tagged PkTRAg proteins for 3 h at room temperature. The plates were then washed and processed for colour development as above.

For antibody inhibition assay, each of the histidine-tagged PkTRAg (250 nM) was pre-incubated with different dilutions of polyclonal sera for overnight at 4°C. This recombinant PkTRAg and anti-PkTRAg antibody reaction mix was then allowed to bind to ~1 million erythrocytes in a 96 well ELISA plates. Plates were then processed for color development as described above after incubating with mouse anti-His_6_ monoclonal antibody. No antibody (only PBS) was taken as control.

### Erythrocyte binding assay by Rosetting

Erythrocytes were collected in 10% citrate phosphate dextrose and were washed three times in RPMI 1640 medium (Invitrogen Life Technologies, Carlsbad CA, USA), pH 7.4, containing 0.36 mM hypoxanthine (Sigma-Aldrich, St. Louis, MO, USA). Erythrocyte binding assay on CHO-K1 cells expressing PkTRAgs on their surface was done as described by Chitnis and Miller [23] and adopted by Zeeshan et al [15]. Erythrocytes at 1% hematocrit in RPMI were added to transfected CHO-K1 cells and incubated for 1 h in a humidified 5% CO_2_ incubator at 37°C. The cells were washed four times with incomplete RPMI 1640 medium, pH 7.4. The numbers of rosettes were scored in 20 fields at 200x magnification. A cluster of five or more erythrocytes bound to a CHO-K1 cell was scored as a rosette. CHO-K1 cells transfected with plasmid pHVDR22 (a kind gift from Dr Chetan Chitnis) encoding PvRII region of Duffy binding protein of *P*.*vivax* [23] and PvTRAg53.7 [15] were used as positive and negative controls, respectively.

For competition assay, human erythrocytes at 1% hematocrit were incubated with purified recombinant proteins at varying concentrations (0–10 μM) in complete RPMI 1640 at room temperature for 1 h. To the transfected CHO-K1 cells, erythrocytes pre-incubated with respective recombinant PkTRAgs were added and rosette formation was observed as above. Results were expressed as relative binding to positive control (erythrocytes binding of PkTRAg transfected cells with untreated RBCs).

For antibody inhibition assay, the CHO-K1 cells transfected with pRE4-PkTRAgs were incubated at 37°C with different dilution of respective anti-PkTRAg sera in RPMI 1640, pH 7.4 for 1 h in a humidified 5% CO_2_ incubator. A pre-immune serum at 1:10 dilution was used as negative control and RPMI 1640 without sera was used as the positive control. Rest of the erythrocytes binding assay was performed as described above. Results are expressed as relative binding to positive control (erythrocytes binding of PkTRAg with no sera).

### *Plasmodium falciparum* growth inhibition assay

Growth inhibition assay was performed as described by Persson et al [26]. Briefly, *Plasmodium falciparum* 3D7 was cultured in complete RPMI 1640 medium containing 27.2 mg/L hypoxanthine, 0.5 g/L Albumax I (Gibco BRL, Grand Island, NY, USA), and 2 g/L sodium bicarbonate, using O^+^ human erythrocytes (4% hematocrit) under mixed gas (5% O2, 5% CO2 and 90% N2). Synchronized parasite cultures at late trophozoite/early schizont stages with 1% parasitemia and 2% hematocrit, were treated with the histidine-tagged recombinant PkTRAgs at a concentration of 20 μM in a 96-well culture plate in triplicate. Uninfected erythrocytes, infected erythrocytes alone and infected erythrocytes with PBS were taken as controls. Parasites were maintained for 48 h and stained with ethidium bromide. One hundred thousand total events were acquired per sample, using FACSDiva software on a BDLSRII flow cytometer (Becton Dickinson Immunocytometry Systems, Palo Alto, USA).

### Statistical Analysis

Unpaired and paired Student’s *t* test and one-way analysis of variance were used to evaluate the statistical significance of these experiments as appropriate. *P* < 0.05 was considered statistically significant. Calculations were performed using graph pad and STATA software.

## Results

### *Plasmodium knowlesi* tryptophan-rich proteins bind to human erythrocytes

**Fig 1:**
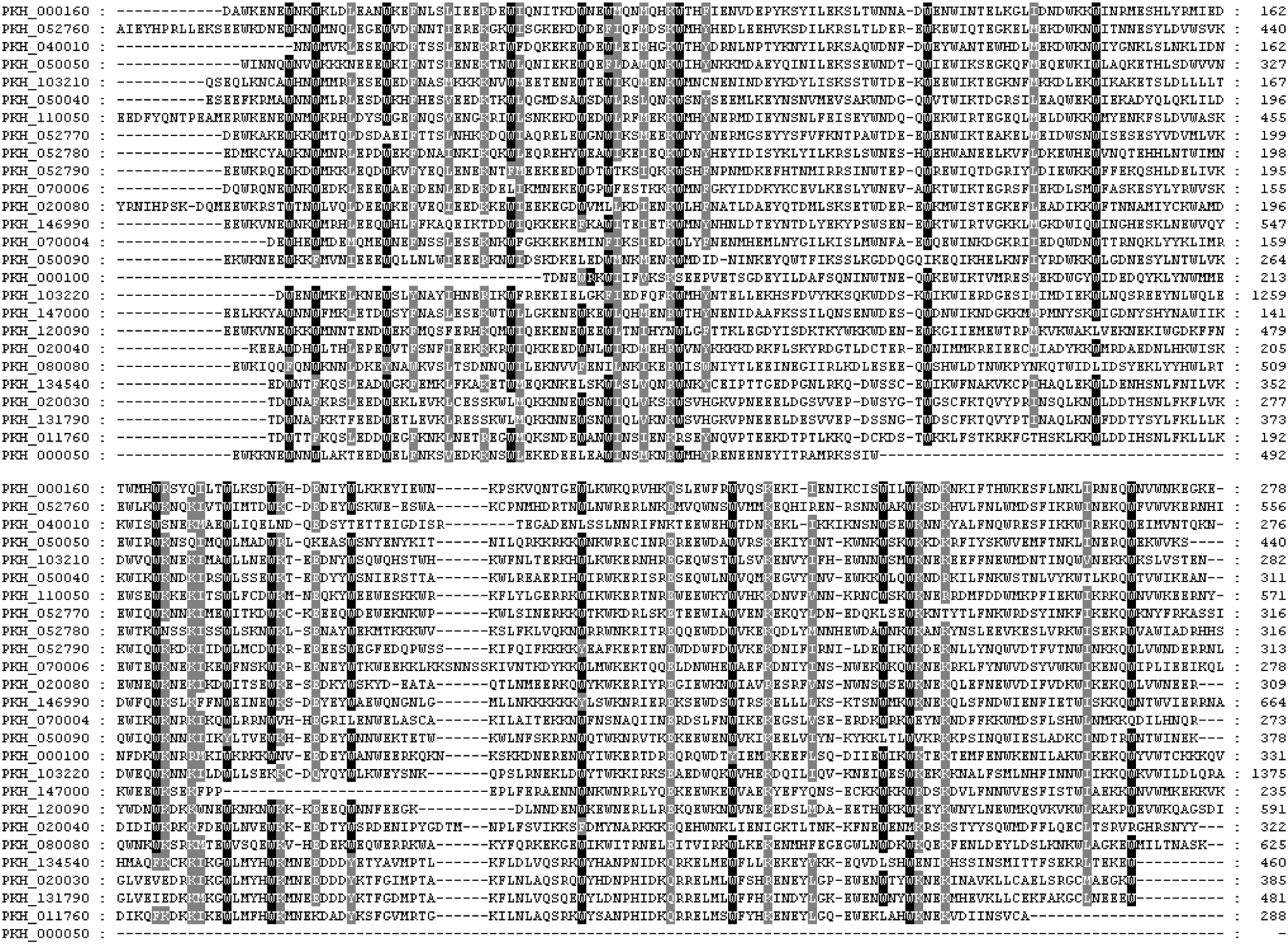
Multiple sequence alignment of *P*. *knowlesi* tryptophan rich antigens (PkTRAgs) using GeneDoc software. *Plasmodium knowlesi* gene sequence retrieval was done at www.Plasmodb.org. Conserved residues are shaded in grey and tryptophan residues are highlighted in black. Position of amino acid residue numbers is shown on the right hand side of the sequence and Plasmodb ID on left hand side. Dashes represent the gaps generated by multiple sequence alignment.

There are twenty six tryptophan-rich antigen encoding genes in *P*.*knowlesi* genome (www.Plasmodb.org). Transcriptome data for 20 of them is available which showed their expression during blood stages of the parasite [20]. Majority of PkTRAg genes contain one intron except PkTRAg181.5 and PkTRAg80.3 which contain two introns while PkTRAg67.1 is intron less (S1 Fig). Exon 1 in most of them is small and mainly coding for the signal peptide. The encoded proteins (PkTRAgs) contain high contents of tryptophan residues (2.1–10.6%) which are positionally conserved (Fig 1). These features of genomic organization and positional conservation of tryptophan residues among PkTRAgs are similar to those described for the ‘Pv-fam-a’ family of *P*.*vivax* proteins (PvTRAgs) probably due to phylogenetic closeness of the two parasite species [11]. The PkTRAgs showed various levels of amino acid sequence homology with tryptophan-rich proteins of *P*.*vivax* (27.9–88.4%), *P*.*falciparum* (23.4–42.4%) and *P*.*yoelii* (23.4–50.1%) (S2 Table).

**Fig 2:**
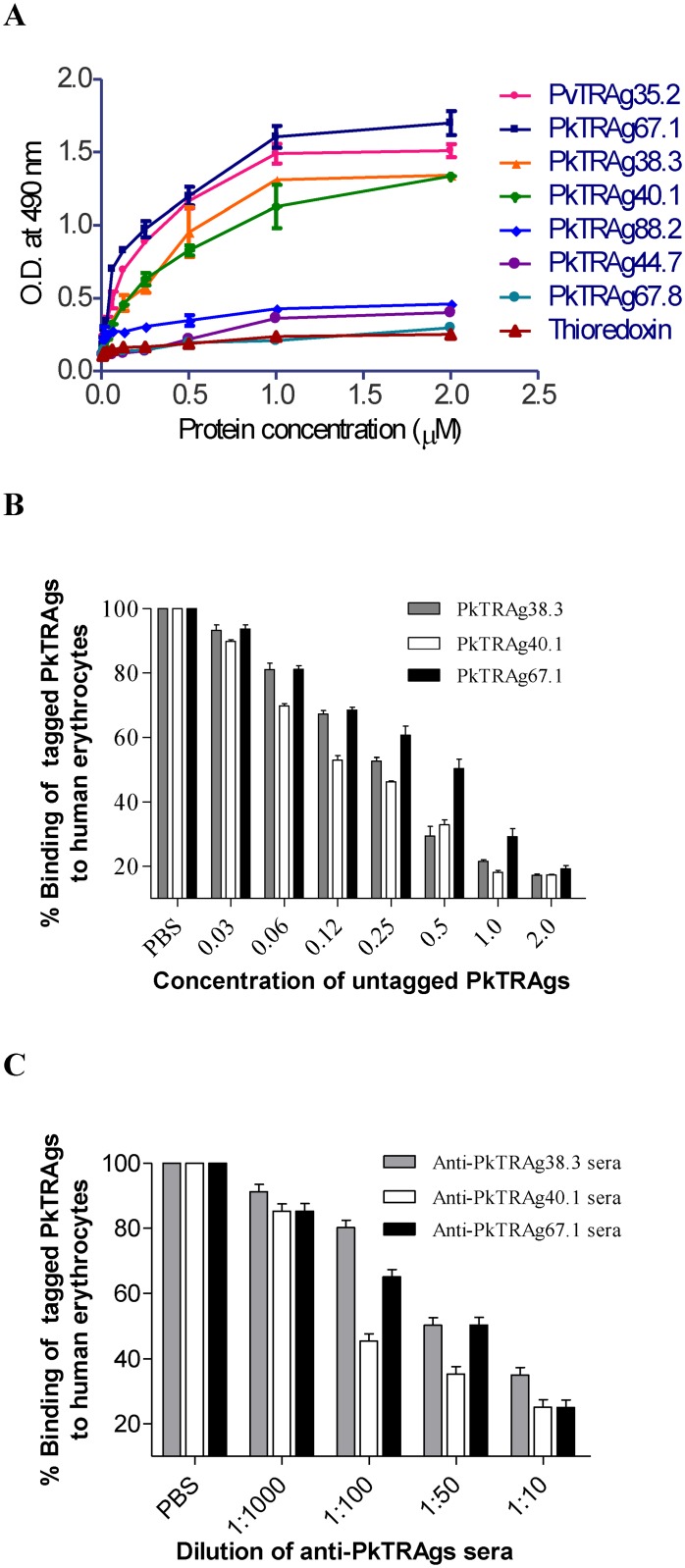
Erythrocyte binding activity of the recombinant PkTRAgs by Cell-ELISA. **(A)** Cell-ELISA. Each well of a microtiter plate containing ~ 1 million of erythrocytes was allowed to interact with different concentration (0–2 μM) of histidine-tagged recombinant proteins. Bacterial thioredoxin and PvTRAg35.2 were used as negative and positive controls, respectively. Plate was developed with monoclonal anti-His_6_ antibody as described in the text. Data shown here are mean ± S.D. of at least three experiments. **(B)** Competition assay. For this assay, 200 nM of each of the recombinant histidine-tagged PkTRAg was mixed with increasing concentration (0–2 μM) of respective untagged PkTRAg. This mixture was then added to the wells of the ELISA plate containing ~ 1 million human erythrocytes. Plate was developed with monoclonal anti-His_6_ antibody as described in the text. Data shown here are mean ± S.D. of at least three experiments. **(C)** Antibody inhibition assay. Different dilutions of polyclonal rabbit sera raised against individual PkTRAg were pre-incubated with the respective recombinant PkTRAg and then added to the well of a microtiter plate containing ~ 1 million human erythrocytes. No antibody i.e. only PBS containing respective histidine-tagged PkTRAg protein was taken as positive control. The plates were developed with mouse anti-His_6_ monoclonal antibody as described in the text. Error bar indicates the standard deviation of mean from three experiments.

We have searched the *P*.*knowlesi* genome sequence with known sequences of PvTRAg proteins which bind to host erythrocytes and selected six close homologues for further characterization. These six PkTRAgs were cloned and expressed here in *E*. *coli*, and these purified recombinant proteins (S2 Fig) were tested for their binding ability to human erythrocytes. The Cell-ELISA results showed that only three of them (PkTRAg38.3, PkTRAg40.1, and PkTRAg67.1) showed this binding activity (Fig 2A). The specificity of binding was confirmed by the competition assay results where histidine-tagged and untagged PkTRAgs competed with each other towards erythrocyte binding in a concentration dependent manner (Fig 2B). This was further confirmed by saturation assay results where excess amount of untagged protein masked the binding sites on human erythrocytes for the respective PkTRAg (data not shown). Furthermore, the respective polyclonal antibody was also able to inhibit the binding of its PkTRAg to human erythrocytes in a dilution dependent manner (Fig 2C).

**Fig 3:**
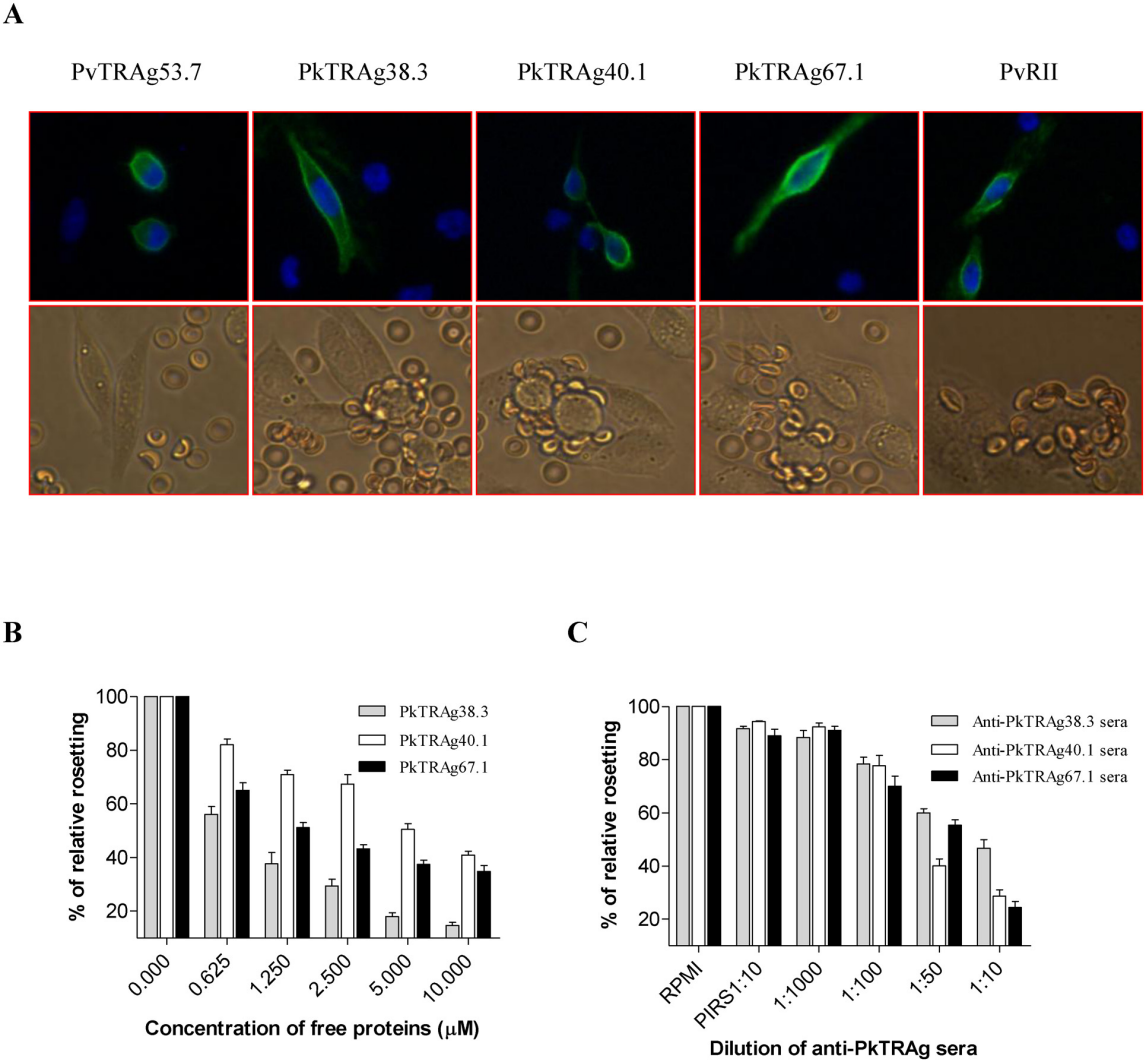
Erythrocyte binding activity of PkTRAgs expressed on the surface of CHO-K1 cells. **(A**) Expression of PkTRAgs on the surface of CHO-K1 cells. The CHO-K1 cells transfected with pRE4-PkTRAgs, pRE4-PvTRAg53.7, and pRE4-PvRII were stained with mouse monoclonal antibodies DL6 directed against C-terminal of the *Herpes simplex* glycoprotein D sequences and then stained with Alexa fluor 488 secondary antibodies. Upper panel shows the merged fluorescence images (Blue for DNA counterstained with DAPI, and green for surface expression of proteins). Lower panel shows the binding of human erythrocytes to the transfected CHO-K1 cells. A single transfected CHO-K1 cell attached with more than five RBCs was considered a rosette. The numbers of rosettes were counted in 20 fields at a 200x magnification. CHO-K1 cells transfected with PvTRAg53.7 and PvRII were used as negative and positive controls, respectively. **(B)** Specificity of PkTRAgs (expressed on surface of CHO-K1 cells) binding to human erythrocytes by competition assay. The human erythrocytes were pre-incubated with different concentrations of PkTRAgs (0–10 μM) before their incubation with transfected CHO-K1 cells to form the rosettes. Erythrocyte binding of CHO cell expressing PkTRAgs with RBCs incubated with PBS only (zero concentration) was taken as positive control. **(C)** Inhibition of erythrocyte binding to PkTRAgs expressed on surface of CHO-K1 cells by polyclonal antibodies. Transfected CHO-K1 cells were pre-incubated with different dilutions of rabbit sera raised against the respective PkTRAg or with pre-immune sera at 1:10 dilution. The percent binding was determined relative to no antibodies (RPMI only). Data shown are the Mean ± standard deviation of three experiments.

Separately, we have also expressed these three PkTRAgs on the surface of mammalian cell line CHO-K1 and used the transfected cells for erythrocyte binding assay to observe rosette formation. The expression of PkTRAg38.3, PkTRAg40.1, and PkTRAg67.1 on CHO-K1 cells surface was confirmed by immunofluorescence assay using monoclonal antibody against HSVgD (Fig 3A, upper panel). Similar surface localization pattern was also observed when a polyclonal rabbit serum against respective PkTRAg was used (Data not shown). The PkTRAg transfected CHO-K1 cells were seen to form the rosettes with uninfected normal human erythrocytes similar to that of the positive control PvRII (Fig 3A, lower panel). Numbers of CHO-K1 cells with rosettes counted in 20 fields at 200 x magnification for PkTRAg67.1, PkTRAg40.1, and PkTRAg38.3 were 16.25 ± 5.7, 11.5 ± 0.5, and 10 ± 0.33, respectively. Under similar conditions, the number of rosettes formed for positive control PvRII and negative control PvTRAg53.7 were 17.8 ± 1.1 and 1.3 ± 0.07, respectively. If the erythrocytes were pre-incubated with different concentrations of the individual PkTRAg and then used for binding to CHO-K1 cell transfected with the respective PkTRAg, the rosette formation was affected (Fig 3B). Similarly, if the CHO-K1 cell expressing the particular PkTRAg was pre-incubated with respective antibody, its rosetting ability with uninfected human erythrocytes was also inhibited in the antibody dilution dependent manner (Fig 3C). Thus, we have confirmed here that PkTRAg67.1, PkTRAg38.3, and PkTRAg40.1 bind to the human erythrocytes and the binding was specific.

### PkTRAg38.3 and PkTRAg40.1 probably recognize the same human erythrocyte receptors as by PvTRAgs of *P*.*vivax*

**Fig 4:**
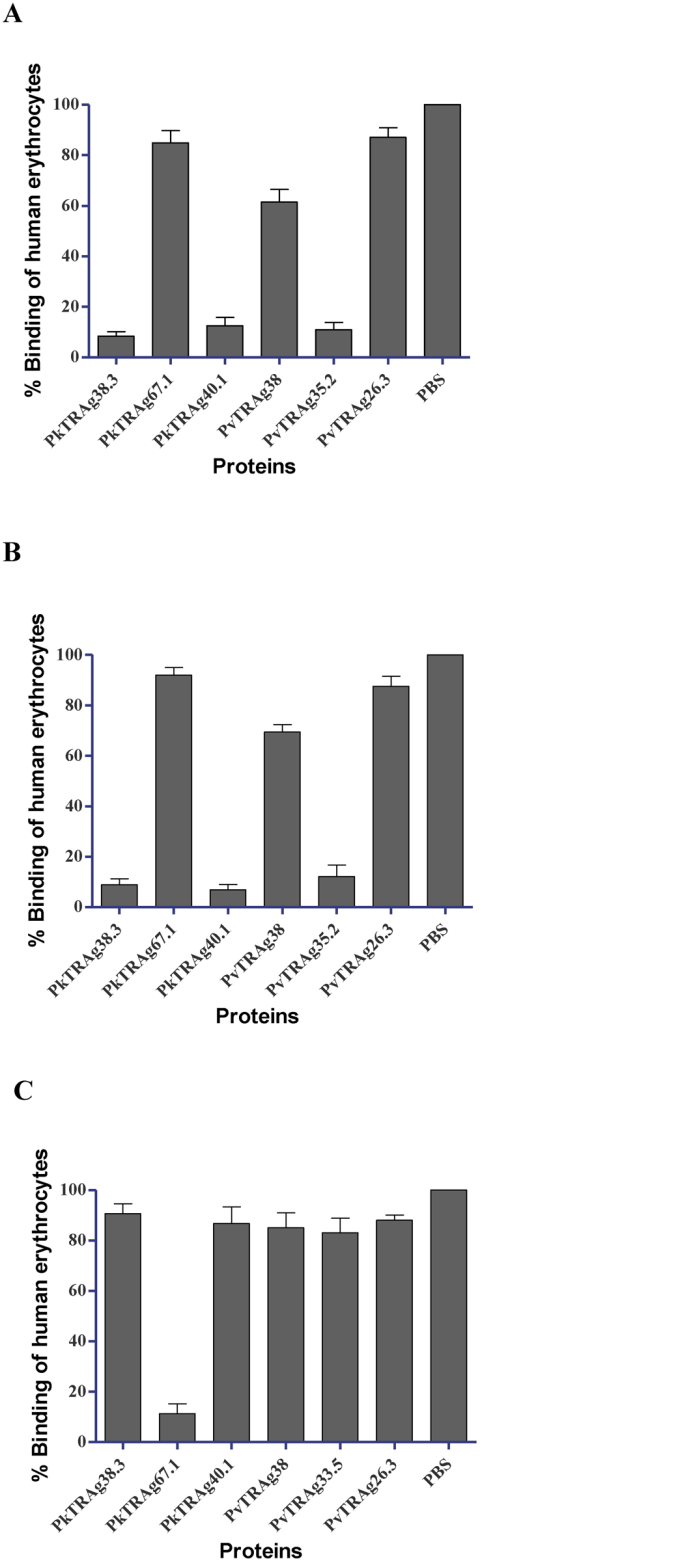
Cross-competition between PkTRAgs and PvTRAgs for erythrocyte binding by Cell-ELISA. Each well of the microtiter ELISA plate containing ~1 million erythrocytes was incubated with 2 μM of untagged PkTRAg38.3, PkTRAg67.1, PkTRAg40.1, PvTRAg38, PvTRAg35.2, or PvTRAg26.3. After washing with PBS, 1 μM of histidine-tagged PkTRAg38.3 **(A)**, PkTRAg40.1 **(B)**, and PkTRAg67.1 **(C)** were added and binding was detected by monoclonal anti-His_6_ antibody as described in text. Mean ± SD value of percent binding of three different experiments is shown. Binding of PkTRAgs without competition (PBS only) was taken as 100%.

A cross-competition assay using Cell-ELISA was performed to investigate if these three binder PkTRAgs were sharing the human erythrocyte receptors between them as well as with PvTRAgs of *P*.*vivax*. Results showed that PkTRAg38.3 and PkTRAg40.1 cross-compete with each other for binding to human erythrocytes (Fig 4A and 4B). These two PkTRAgs also showed cross-competition with previously described PvTRAg35.2. These two PkTRAgs did not compete with PkTRAg67.1 and PvTRAg26.3 and thus do not share their erythrocyte receptors. However, there was a partial competition between PvTRAg38 and these two PkTRAgs for erythrocyte binding indicating that only one of the receptor is common for these parasite ligands (Fig 4A and 4B).

### PkTRAg67.1 binds to different erythrocyte receptor(s)

PkTRAg67.1 did not cross-compete with other two PkTRAgs for erythrocyte binding (Fig 4C). However, we noticed that PkTRAg67.1 also did not cross-compete for the erythrocyte binding with any of the previously described *P*. *vivax* proteins (PvTRAg35.2, PvTRAg38, and PvTRAg26.3) covering the entire spectrum of erythrocyte binders (Fig 4C). These results indicate that PkTRAg67.1 was recognizing a different set of erythrocyte receptors than described so far for PvTRAgs.

### PkTRAgs inhibit the *P*. *falciparum* parasite growth in in-vitro culture

**Fig 5:**
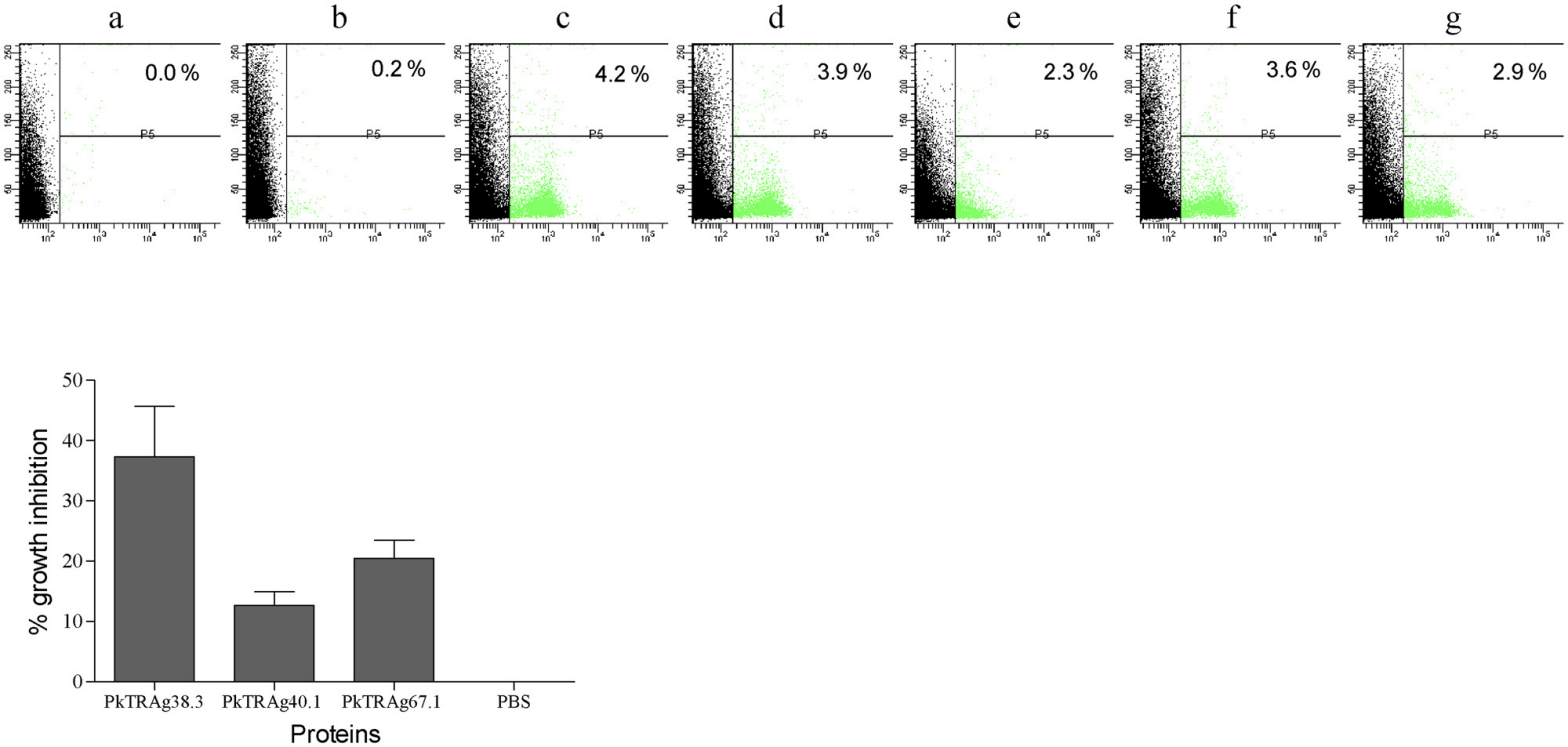
PkTRAgs inhibit *P*.*falciparum* growth in in-vitro culture. Parasite culture at late trophozoite/early schizont stage was incubated with 20 μM of PkTRAg38.3, PkTRAg40.1, PkTRAg67.1 and other controls. After 48 h, parasitemia was determined by ethidium bromide staining and measured by flowcytometry. Representative dot plots are shown in upper panel; **a**. unstained erythrocytes, **b**. uninfected erythrocytes, **c**. infected erythrocytes, **d**. infected erythrocytes with PBS, **e**. infected erythrocytes with PkTRAg38.3, **f**. infected erythrocytes with PkTRAg40.1, and **g**. infected erythrocytes with PkTRAg67.1. Bar diagram shows the percentage of parasite growth inhibition. The data is depicted as a mean ± SD for two triplicate experiments.

In order to understand the biological significance of erythrocyte binding activity of these PkTRAgs, we added these *P*.*knowlesi* proteins to the *P*.*falciparum* culture. All three erythrocyte binding PkTRAgs were able to inhibit the *P*.*falciparum* parasite growth albeit at a different rate (Fig 5). The parasite growth inhibitory effect of PkTRAg67.1 was interesting since this *P*.*knowlesi* molecule recognized different erythrocyte receptors which are not shared by PkTRAg38.3 and PkTRAg40.1 or by any of the previously characterized PvTRAgs. Surprisingly, there was a difference in the parasite growth inhibition rates by PkTRAg38.3 and PkTRAg40.1, although both of them compete with each other in erythrocyte binding assays, requires further investigations. It is possible that these two PkTRAgs are not recognizing same erythrocyte receptors during cross-competition but binding of one PkTRAg is physically blocking the binding of other to a nearby receptor which may be different.

## Discussion

Zoonotic parasite, *P*. *knowlesi* has now emerged as an important human malaria causing species [1]. This host switching ability of *P*.*knowlesi* should involve interaction between proteins of this parasite and human erythrocytes [4, 5]. We have recently described the interaction between PvTRAgs of *P*.*vivax* and human erythrocytes [15], and wanted to know if PkTRAgs of *P*.*knowlesi* would also recognize the human erythrocyte receptors for host-parasite interaction similar to that of the PkNBPXa which may lead to this zoonotic disease [5]. We have selected 6 PkTRAgs which were close homologues of recently described PvTRAgs and found that three of them (PkTRAg38.3, PkTRAg40.1 and PkTRAg67.1) were binding to human erythrocytes. This binding was confirmed by more than one assay and found to be specific by competition assays (Figs 2 and 3). Binding of these PkTRAgs with monkey erythrocytes need to be investigated in order to find out if some of these proteins bind to human as well as monkey erythrocytes as noted for *P*.*knowlesi* ligand PkNBPXa [5]. Similar to tryptophan-rich proteins of *P*.*yoelii*, *P*.*falciparum*, and *P*.*vivax*, which have been described to be associated with host cell invasion phenomenon [13, 15, 27], it is possible that this receptor-ligand interaction between human erythrocyte molecules and PkTRAgs is also associated with similar phenomenon during malaria zoonosis. It may be stated here that only fewer *P*.*knowlesi* proteins and their interacting partners on human erythrocyte are known, so far [4, 28].

The transcriptome data for PkTRAgs [20] shows that erythrocyte binding proteins PkTRAg 40.1 and PkTRAg 67.1 are expressed at the late stage of the parasite (expression profile of PkTRAg38.3 is not available) therefore could be associated with red cell invasion process. This is indeed supported by our in-vitro *P*.*falciparum* growth inhibition assay results (Fig 5) where all the three erythrocyte binding PkTRAgs affected the parasite growth. It may be interesting to note that PkTRAg38.3 and PkTRAg67.1 do not have a signal peptide or the transmembrane domain but probably transported to parasite or infected erythrocyte surface for erythrocyte binding. But this is not surprising as there are other *Plasmodium* proteins which lack such signal sequences and still transported from the parasite [29]. This could well be an indication that parasite uses yet another unknown mechanism to export some of its proteins. Although this could also be a problem of genome annotation, localisation studies with tagged parasites or IFAs need to be carried out to demonstrate that these specific proteins are secreted/exported to the parasite or iRBC surface.

We wanted to investigate if these three PkTRAgs were sharing the human erythrocyte receptors with each other or not. The cross-competition results indicate that PkTRAg38.3 and PkTRAg40.1 were competing with each other for the same erythrocyte receptor(s) while PkTRAg67.1 was recognizing a separate set of erythrocyte receptors (Fig 4). However, cross-competition between PkTRAg38.3 and PkTRAg40.1 could also occur if binding of one ligand physically blocks the binding of the second ligand to a nearby but different receptor. Or in a scenario of two erythrocyte receptors for each PkTRAg, similar to that of PvTRAgs [14, 15], it is possible that binding of ligands to the common receptor makes their respective second receptor (which may be different) inaccessible due to physical blocking or steric hindrance. These three PkTRAgs could be recognizing four or more different types of human erythrocyte receptors similar to our earlier observations on *P*.*vivax* PvTRAgs where each protein recognized two erythrocyte receptors and same erythrocyte receptor was recognized by more than one protein [14, 15].

Next, we were interested to find out if these PkTRAgs were sharing their human erythrocyte receptors with previously described PvTRAgs of *P*.*vivax*. For this, the cross-competition assays between PkTRAgs and PvTRAgs (PvTRAg26.3, PvTRAg38 and PvTRAg35.2) for erythrocyte binding were performed. These three PvTRAgs covered the entire spectrum of five erythrocyte receptors recognized by all ten PvTRAgs [14, 15]. Both PkTRAg38.3 and PkTRAg40.1 recognized the same erythrocyte receptors that were recognized by PvTRAg35.2. (Fig 4) Indeed, PvTRAg35.2 recognizes two erythrocyte receptors (receptor ‘A’ and ‘B’) which were also recognized by four other PvTRAgs (PvTRAg33.5, PvTRAg, PvTRAg69, and PvTRAg34) [15]. As mentioned above, the possibility of only one of the two receptors being common to these proteins cannot be ruled out. This indicates that these two human erythrocyte receptors are being recognized by the maximum number of *Plasmodium* tryptophan-rich proteins. These two PkTRAgs were also partially competing with PvTRAg38 for human erythrocyte receptors suggesting the involvement of one common receptor between them. Surprisingly, PkTRAg67.1 did not cross-compete with other two PkTRAgs as well as with any of the PvTRAgs including PvTRAg26.3 which recognizes yet another set of erythrocyte receptors [15] (Fig 4). This indicates that PkTRAg67.1 must be recognizing the different receptor molecule(s) on human erythrocyte which has not been recognized by any other PvTRAg or PkTRAg, described so far. Although similar cross-competition data for *P*.*falciparum* tryptophan-rich antigens is awaited, the inhibitory effect of these PkTRAgs on the *P*.*falciparum* parasite growth in in-vitro culture system (Fig 5) seems to suggest that these PkTRAgs are sharing the human erythrocyte receptors with *P*.*falciparum* molecules. Therefore, PkTRAg67.1 could be recognizing the same erythrocyte receptors which are also utilized by the *P*.*falciparum* ligands but not by PvTRAgs. These results also indicate the biological significance of PkTRAgs in progression of zoonotic disease caused by *P*.*knowlesi*.

In conclusion, three tryptophan rich proteins of monkey malaria parasite PkTRAg38.3, PkTRAg40.1 and PkTRAg67.1 exhibit the human RBC binding activity. These proteins could have their roles in rosetting for disease pathogenesis or be associated with red cell invasion process. Two of these proteins, PkTRAg38.3, PkTRAg40.1, shared the most commonly used human erythrocyte receptors as by majority of the PvTRAgs of its natural parasite *P*.*vivax*. The parasite growth inhibitory effect of these PkTRAgs in *P*.*falciparum* culture could also be an indication that ligands of these two parasite species are also sharing the human erythrocyte receptors with each other. Sharing of human erythrocyte receptors by monkey and human malaria parasites described here is probably allowing the *P*.*knowlesi* to establish itself inside the heterologous host and leading to this zoonotic disease.

## Supporting Information

S1 Fig Schematic representation of introns and exons of PkTRAg genes. Based on the number of exons, PkTRAgs are divided in to three groups; (A) single exon, (B) two exons, and (C) three exons. Exon and intron are shown by boxes and lines, respectively, and their sizes in base pairs are shown on top of lines or boxes. The tryptophan-rich domain is shaded in grey and encoded amino acid length of the domain is given below the box. The predicted signal peptide (SP) and transmembrane domain (TM) are shown in exon 1. The total number of amino acid residues present in each PkTRAg along with % tryptophan contents is shown on the right hand side. Accession number and name of the respective PkTRAg in bracket is shown on left hand side. #Nomenclature is done according to the molecular weights prefixed by PkTRAg. The sequences of these antigens are retrieved from PlasmoDB database (www.Plasmodb.org) and analyzed.
(TIF)

S2 Fig SDS-PAGE profile of purified recombinant histidine-tagged PkTRAgs. Lane 1, PkTRAg38.3; lane 2, PkTRAg40.1; lane 3, PkTRAg44.7; lane 4, PkTRAg67.1; lane 5, PkTRAg67.8; lane 6, PkTRAg88.2. Size of molecular weight markers is indicated in left hand side.
(TIF)

S1 Table Primer sequences and PCR conditions for the amplification of PkTRAgs genes. (DOCX)

S2 Table Homology of *P*.*knowlesi* tryptophan- rich antigens with the tryptophan rich proteins from other *Plasmodium* species. (DOC)
