# Supplementary figures and images for: Recognition of Human Erythrocyte Receptors by the Tryptophan-Rich Antigens of Monkey Malaria Parasite Plasmodium knowlesi
Source: PLoS One. 2015 Sep 22;10(9):e0138691. doi: 10.1371/journal.pone.0138691 (PMC4579084; doi:10.1371/journal.pone.0138691)

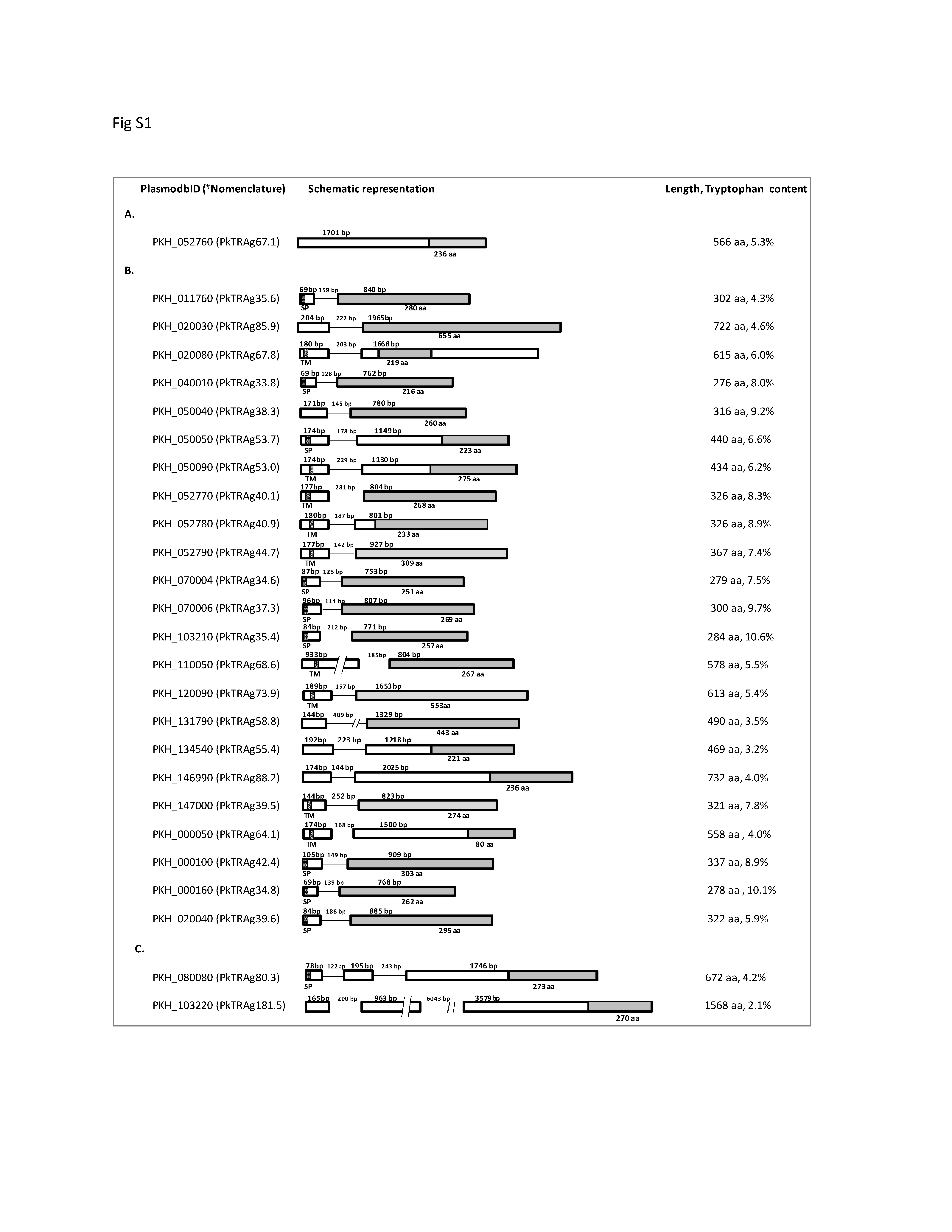

Supplement: S1 Fig — Based on the number of exons, PkTRAgs are divided in to three groups; (A) single exon, (B) two exons, and (C) three exons. Exon and intron are shown by boxes and lines, respectively, and their sizes in base pairs are shown on top of lines or boxes. The tryptophan-rich domain is shaded in grey and encoded amino acid length of the domain is given below the box. The predicted signal peptide (SP) and transmembrane domain (TM) are shown in exon 1. The total number of amino acid residues present in each PkTRAg along with % tryptophan contents is shown on the right hand side. Accession number and name of the respective PkTRAg in bracket is shown on left hand side. #Nomenclature is done according to the molecular weights prefixed by PkTRAg. The sequences of these antigens are retrieved from PlasmoDB database (www.Plasmodb.org) and analyzed. (TIF) [file pone.0138691.s001.tif]

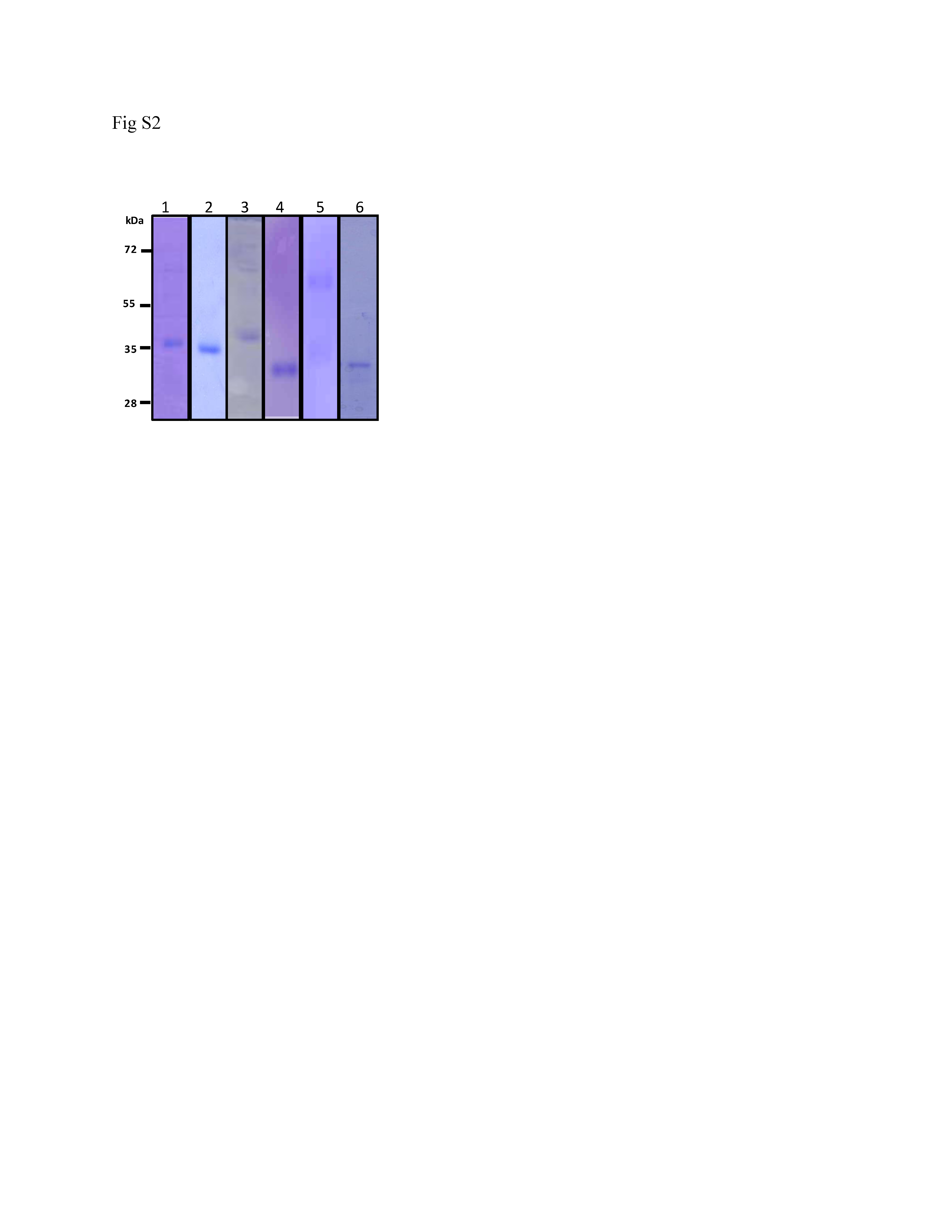

Supplement: S2 Fig — Lane 1, PkTRAg38.3; lane 2, PkTRAg40.1; lane 3, PkTRAg44.7; lane 4, PkTRAg67.1; lane 5, PkTRAg67.8; lane 6, PkTRAg88.2. Size of molecular weight markers is indicated in left hand side. (TIF) [file pone.0138691.s002.tif]
