# Supplementary material for: Recognition of Human Erythrocyte Receptors by the Tryptophan-Rich Antigens of Monkey Malaria Parasite Plasmodium knowlesi
Source: PLoS One. 2015 Sep 22;10(9):e0138691. doi: 10.1371/journal.pone.0138691 (PMC4579084; doi:10.1371/journal.pone.0138691)
Supplement: S1 Table — (DOCX) [file pone.0138691.s003.docx]

S1 Table. Primer sequences and PCR conditions for the amplification of PkTRAgs genes.

| Gene name | Primer name | Primer Sequence | PCR conditions* |
| --- | --- | --- | --- |
| PkTRAg44.7 | **Primary PCR**  90 exfor1  90exrev1  **Nested PCR**  90exfor2  90exrev2 | 5’-GTGTCAGAATGGAGATATTAAGGG-3’  5’-GTGTCCAATGAGGAACGCTAAGAG-3’  5’-CGATGATGAGTCGACCCCAAATGAG-3’  5’-TGTTTCTCGAGATCACTTTTCATT-3’ | Annealing at 57^0^ C for 1 min,  Extension at 68^0^ C for 40 sec.  Annealing at 62^0^ C for 1 min,  Extension at 68^0^ C for 40 sec. |
| PkTRAg38.3 | **Primary PCR**  40 ex for1  40 ex rev1  **Nested PCR**  40 ex for2  40 ex rev2 | 5-’CCGGAATACCAATTAATCCTCATA-3’  5’GCTTTCATTCATTATTAGAATGCC3’  5’-TCCTATCTAGGATCCACCTAATAGA-3’  5-’AAGTTTAAGCTTCTAAACTTTATTTT-3’ | Annealing at 56^0^ C for 1 min,  Extension at 68^0^ C for 1 min.  Annealing at 55^0^ C for 1 min,  Extension at 68^0^ C for 1 min. |
| PkTRAg67.1 | **Primary PCR**  60 for1  60 rev1  **Nested PCR**  60 exfor2  60 exrev3 | 5’- CTACCAACCTCTAGCCCATTTCCC-3’  5’-GTCGCGGTGTCCAGTGGTTCTTGT-3’  5’- ATGGATCCATACTATAAAGAAGTG-3’  5’-TTATTTGAAGCTTTTATATCTTCC-3’ | Annealing at 67^0^ C for 1 min,  Extension at 68^0^ C for 2 min.  Annealing at 50^0^ C for 1 min,  Extension at 68^0^ C for 2 min. |
| PkTRAg40.1 | **Primary PCR**  70 for1  70 rev1  **Nested PCR**  70 exfor2  70 exrev2 | 5’- ATGGAAGTAGTTCCTGAAGCACCA- 3’  5’- CTAATTAAAGCTTAACATAGATGC-3’  5’-CCGTCGACGAAAAAAAAAT T AAAG-3’  5’- AGC TCGAGGCTAATTACTGATGAA-3’ | Annealing at 58^0^ C for 1 min,  Extension at 68^0^ C for 1 min.  Annealing at 50^0^ C for 1 min,  Extension at 68^0^ C for 1 min. |
| PkTRAg88.2 | **Primary PCR**  146 ex for1  146 ex rev1  **Nested PCR**  146 ex for2  146 ex rev2 | 5’- CTTCTCAATTGATCTAAAAATCGC- 3’  5’ -CAACAAATTACAAGGTGATAATCC -3’  5’-AAAAAGGATCCAACAGAAGAATGG-3’  5’-AAGCTTAATATGAAGGTGATTTTTTTC-3’ | Annealing at 55^0^ C for 1 min,  Extension at 68^0^ C for 2:30 min.  Annealing at 53^0^ C for 30 sec,  Extension at 68^0^ C for 1 min. |
| PkTRAg67.8 | **Primary PCR**  80exfor1  80 exrev1  **Nested PCR**  80 for2  80 rev2 | 5’-ATTTATACATATACGTATTTAACA-3’  5’ -TTTGCCTGCGAATGCTC TTTTGC-3’  5’-TCAGAGGATCCATACGCCAGGTCG-3’  5’-TTTAAGCTTCTTAACTACCAAGGG-3’ | Annealing at 55^0^ C for 1 min,  Extension at 68^0^ C for 2:30 min.  Annealing at 57^0^ C for 1 min,  Extension at 68^0^ C for 2:30 min. |

The primers for PkTRAg genes were designed based on sequence of *Plasmodium knowlesi*, strain H (www.plasmodb.org). Restriction sites in primer sequences are underlined.

*Initial denaturation was done at 94^0^ C for 10 minutes and subsequent denaturation at 94^0^ C for 30 seconds. The final extension was at 68^0^ C for 10 minutes. A total of 35 cycles for primary and 25 cycles for nested PCR were performed.
