# Supplementary material for: Recognition of Human Erythrocyte Receptors by the Tryptophan-Rich Antigens of Monkey Malaria Parasite Plasmodium knowlesi
Source: PLoS One. 2015 Sep 22;10(9):e0138691. doi: 10.1371/journal.pone.0138691 (PMC4579084; doi:10.1371/journal.pone.0138691)
Supplement: S2 Table — (DOC) [file pone.0138691.s004.doc]

S2 Table. Homology of *P.knowlesi* tryptophan- rich antigens with the tryptophan rich proteins from other *Plasmodium* species.

| **S. No.** | ***P.knowlesi* Proteins** | **Homology with *P.vivax* tryptophan rich proteins** | | | **Homology with *P.falciparum* tryptophan rich proteins** | | | **Homology with *P.yoelii* tryptophan rich proteins** | | |
| --- | --- | --- | --- | --- | --- | --- | --- | --- | --- | --- |
|  |  | **Protein** | **% homology (Identity)** | **overlap (aa)** | **Protein** | **% homology (Identity)** | **overlap (aa)** | **Protein** | **% homology (Identity)** | **overlap (aa)** |
| 1 | PkTRAg67.1 | PvTRAg39.8 | 95 (88) | 268 | PfTryThrA | 64 (41) | 232 | PypAg-3 | 71 (50) | 269 |
| 2 | PkTRAg38.3 | PvTRAg38 | 87 (77) | 316 | PfTryThrA | 65 (39) | 233 | PypAg-1 | 59 (33) | 232 |
| 3 | PkTRAg53 | PvTRAg56.2 | 70 (59) | 399 | PfTryThrA | 61 (61) | 270 | PypAg-3 | 55 (35) | 213 |
| 4 | PkTRAg67.8 | PvTRAg55 | 80 (64) | 320 | PfTryThrA | 55 (32) | 242 | PypAg-1 | 59 (38) | 244 |
| 5 | PkTRAg88.2 | PvTRAg74 | 75 (62) | 350 | PfTryThrA | 52 (31) | 323 | PypAg-3 | 54 (33) | 235 |
| 6 | PkTRAg40.1 | PvTRAg39.9 | 85 (75) | 326 | PfTryThrA | 60 (35) | 256 | PypAg-3 | 60 (35) | 225 |
| 7 | PkTRAg68.6 | PvTRAg69.4 | 63 (46) | 599 | PfTryThrA | 56 (37) | 227 | PypAg-3 | 57 (32) | 242 |
| 8 | PkTRAg37.3 | PvTRAg55 | 53 (30) | 254 | PfTryThrA | 51 (34) | 255 | PypAg-3 | 54 (31) | 227 |
| 9 | PkTRAg35.4 | PvTRAg38.7 | 88 (79) | 284 | PfTryThrA | 54 (34) | 254 | PypAg-1 | 58 (35) | 222 |
| 10 | PkTRAg53.7 | PvTRAg53.7 | 70 (61) | 384 | PfTryThrA | 56 (35) | 198 | PypAg-1 | 53 (29) | 202 |
| 11 | PkTRAg40.9 | PvTRAg 42.9 | 76 (58) | 326 | PfTryThrA | 48 (31) | 266 | PypAg-3 | 54 (31) | 250 |
| 12 | PkTRAg34.8 | PvTRAg35.2 | 86 (75) | 270 | PfTryThrA | 57 (34) | 226 | PypAg-1 | 54 (33) | 239 |
| 13 | PkTRAg44.7 | PvTRAg | 72 (61) | 311 | PfTryThrA | 55 (35) | 236 | PypAg-3 | 55 (28) | 237 |
| 14 | PkTRAg33.8 | PvTRAg33.6 | 81 (74) | 276 | PfTryThrA | 49 (31) | 241 | PypAg-3 | 46 (28) | 261 |
| 15 | PkTRAg181.5 | PvTRAg309 | 65 (53) | 902 | PfTryThrA | 53 (30) | 217 | PypAg-3 | 57 (30) | 216 |
| 16 | PkTRAg80.3 | PvTRAg99.6 | 73 (59) | 372 | Trp-3 | 55 (29) | 232 | PypAg-3 | 48 (26) | 226 |
| 17 | PkTRAg42.4 | PvTRAg37.4 | 81 (66) | 294 | PfTryThrA | 51 (28) | 266 | PypAg-3 | 48 (24) | 273 |
| 18 | PkTRAg73.9 | PvTRAg55 | 41 (26) | 254 | LysTrpA | 48 (29) | 215 | PypAg-3 | 39 (23) | 234 |
| 19 | PkTRAg34.6 | PvTRAg34.9 | 86 (75) | 244 | PfTryThrA | 49 (28) | 239 | PypAg-3 | 48 (27) | 226 |
| 20 | PkTRAg39.6 | PvTRAg39.8a | 83 (72) | 314 | PfTryThrA | 50 (30) | 175 | PypAg-3 | 50 (27) | 213 |
| 21 | PkTRAg39.5 | PvTRAg40 | 67 (55) | 259 | PfTryThrA | 44 (26) | 227 | PypAg-3 | 45 (27) | 227 |
| 22 | PkTRAg55.4 | PvTRAg157 | 73 (52) | 229 | PfTryThrA | 45(24) | 225 | PypAg-1 | 45 (23) | 226 |
| 23 | PkTRAg58.8 | PvTRAg157 | 62 (40) | 284 | PfTryThrA | 48 (26) | 235 | PypAg-3 | 43 (24) | 234 |
| 24 | PkTRAg64.1 | PvTRAg80.6 | 63 (52) | 493 | PfTryThrA | 55 (30) | 131 | PypAg-1 | 60 (35) | 76 |
| 25 | PkTRAg85.9 | PvTRAg154 | 70 (54) | 219 | PfTryThrA | 46 (23) | 179 | PypAg-1 | 47 (26) | 201 |
| 26 | PkTRAg35.6 | PvTRAg38.7 | 63 (49) | 219 | PfTryThrA | 40 (21) | 179 | PypAg-1 | 42 (23) | 201 |

Composition-based homology search was carried out by sequence alignment using BLASTP 2.2.17. PfTryThrA: *P.falciparum* tryptophan threonin rich antigen, Trp-3; Tryptophan rich protein-3, LysTrpA ; Lysineand tryptophan rich antigen, PypAg: *P.yoelii* secreted blood-stage antigen, aa: amino acid.
